# Testing Different Versions of the Affective Neuroscience Personality Scales in a Clinical Sample

**DOI:** 10.1371/journal.pone.0109394

**Published:** 2014-10-07

**Authors:** Pedersen Geir, Johansen Merete Selsbakk, Wilberg Theresa, Karterud Sigmund

**Affiliations:** 1 Department of Personality Psychiatry, Clinic of Mental Health and Addiction, Oslo University Hospital, Oslo, Norway; 2 Department of Research and Development, Clinic of Mental Health and Addiction, Oslo University Hospital, Oslo, Norway; 3 Institute of Clinical Medicine, Faculty of Medicine, University of Oslo, Oslo, Norway; University of Stellenbosch, South Africa

## Abstract

**Background:**

As a tool to investigate the experiences of six primary emotions, Davis, Panksepp, and Normansell [1] developed the Affective Neuroscience Personality Scales (ANPS). However, the psychometric properties of the ANPS have been questioned, and in particular the factor structure. This study replicates earlier psychometric studies on ANPS in a sample of (546) personality disordered patients, and also includes ANPS-S, a recent short version of ANPS by Pingault and colleagues [2], and a truncated version of BANPS by Barrett and colleagues [3].

**Methodology/Principal Findings:**

The study of the full ANPS revealed acceptable internal consistencies of the primary emotion subscales, ranging from 0.74–0.87. However, factor analyses revealed poor to mediocre fit for a six factor solution. Correlational analyses, in addition, revealed too high correlations between PLAY and SEEK, and between SADNESS and FEAR. The two short versions displayed better psychometric properties. The range of internal consistency was 0.61–0.80 for the BANPS scales and 0.65–84 for the ANPS-S. Backward Cronbach Alpha Curves indicated potentials for improvement on all three versions of the questionnaire. Items retained in the short versions did not systematically cover the full theoretical content of the long scales, in particular for CARE and SADNESS in the BANPS. The major problems seem to reside in the operationalization of the CARE and SADNESS subscales of ANPS.

**Conclusions/Significance:**

Further work needs to be done in order to realize a psychometrically sound instrument for the assessment of primary emotional experiences.

## Introduction

Since the writings of Hippocrates (460-370 BC), temperament has been regarded as an important source of variation accounting for differences in personality between people. Temperament has always been regarded as highly “constitutional”. This view has been supported by modern heredity studies, which have found heritability to be in the range of .40–.50 for personality traits [4] and .20–.50 for personality disorders (PDs) [5]. Individual differences in temperament are supposed to reflect different trigger thresholds, intensities, and regulatory mechanisms of emotions [6]. In past decades, theories of temperament have been enriched by theories of primary emotional systems. It is argued that these emotional systems have been shaped by evolution to sustain life, and to enhance reproduction and care for offspring [7], [8]. It is important to note that emotional systems are conceived as complex behavioral systems that affect the organism as a whole, while feelings are considered the conscious awareness of the organism being emotionally aroused [8].

There are some disagreements regarding what should count as primary emotional systems among animals [9], [10], [11], [12], [13]. The most comprehensive theory, based on animal models, has been conceived by Panksepp [12], [13], who included the following seven emotions: PLAY (playfulness), SEEK (seeking), CARE (caring), LUST (sexual), FEAR, ANGER, and SADNESS.

The hypothesis that individual differences with respect to primary emotions have a significant impact upon personality and PDs, has important implications for research. One important step in testing this hypothesis has been the construction of a self-report questionnaire that aims to assess the conscious feelings and behavioral tendencies of being aroused by primary emotions. Based on Spielberger’s work on the State-Trait Personality Inventory (STPI) [14], Davis, Panksepp, and Normansell [1] developed The Affective Neuroscience Personality Scales (ANPS). The likelihood of its success in advancing the field of personality differences and PDs depends on its construct validity, which includes the content validity and internal consistency of its operationalizations, the overall structure of the instrument, its relations to other social and clinical constructs, and the incremental validity it represents by assessing individual variation not covered by other traditional measures and measurement methods.

The initial version of ANPS [1] contained 110 items. The ANPS has since been revised by 33 items, and has increased from 110 to 112 items in total (ANPS version 2.4) [15]. The questionnaire includes six of the seven primary emotions as defined by Panksepp [12]. LUST is not included in the ANPS, because the authors suspected that it might be an emotion that people would be less frank about ([15]; pp. 1949). An operationalization of a construct called Spirituality is also implemented in the ANPS. Spirituality is believed to be clinically important in the treatment of alcoholism [16], [17], [18]. Sixteen additional filler items are included. The authors selected these items to represent three additional subscales, merely for personal interests [1], called Dominance, Social Anxiety, and Unlikely Virtue. The latter is composed as a traditional ‘validity scale’, with an expected low variance. In this article, we will focus on the six “true” primary emotions only.

Previous studies of the ANPS have mainly involved student populations. Davis and colleagues [1] studied the psychometric properties and associations to the five-factor model of personality (FFM) [19] in two American samples: college students in psychology (n = 171, 71% female, mean age 20 years), and job applicants (n = 598, 18% female, mean age 42 years). Pahlavan and coworkers [20] used a sample of 412 French college students (77% female, mean age 20 years). Pingault and coworkers [2], [21] used a sample of 830 French students/young adults (55% female, mean age 21 years). Abella and coworkers [22] analyzed a sample of 402 Spanish students (55% female, mean age 23 years), while Barrett and coworkers [3] studied 2,821 college students.

In all, the internal consistencies of the revised ANPS have proven acceptable in the studies of Pahlavan, Pingault, and Abella, with Cronbach’s alpha coefficient [23] ranging between .70 and .90. However, in the study by Pingault et al. [21], the SEEK scale had an alpha of .64. Another area of concern has been high intercorrelations between the SADNESS and the FEAR subscales.

Some consistent gender differences have been observed among validity studies of the ANPS. Men tend to score higher than women on the PLAY scale, while women tend to score higher than men on the FEAR, SADNESS, and CARE scales. However, the magnitude of the observed differences has been small.

The most serious shortcoming of ANPS is probably that most studies have failed to demonstrate a valid six factor solution by confirmatory factor analyses. This fact, combined with overly long scales, some poorly worded items and extended overlap between some subscales (FEAR and SADNESS), have motivated investigators to construct short forms of the ANPS. Pingault and coworkers [2] extracted the 6 best functioning items for each subscale into a 36 items short form (ANPS-S). Based on a sample of 850 young French participants and 431 young to middle aged Canadian participants, they found that the factor structure of the ANPS-S fitted the theoretical structure of the instrument better than the long version. Other psychometric properties were also satisfactory. Barrett and colleagues [3] constructed another brief form (BANPS), consisting of 33 items whereof 5 items were created anew. Based on three separate studies, comprising 439, 738 and 1096 students, respectively, they demonstrated a clear and coherent factor structure by this brief form, as well as other enhanced psychometric properties.

Since the populations on which the psychometric studies of ANPS have been questioned, mostly consisted of college students, there is a need to investigate if the same limitations hold true also for a clinical population where the distribution of the different primary emotions are different, and if the short versions exhibit better psychometric properties also for such a population.

The aim of the present study was to replicate earlier psychometric studies on ANPS, ANPS-S, and a truncated version of BANPS in a clinical sample. The participants in this study were patients with personality disorders who are known to vary considerably with respect to primary emotions such as FEAR, CARE, SADNESS and ANGER.

## Materials and Methods

### The Sample

This multi-site study comprised data from 546 patients consecutively admitted to five different treatment units participating in the Norwegian Network of Personality-Focused Treatment Programs [24] from January 2004 to May 2013. The majority of patients were female (77%) and the mean age was 32 years (SD = 8). All patients were diagnosed according to the DSM-IV [25] by use of the Structured Clinical Interview for DSM-IV Axis II Personality Disorders (SCID-II) [26], according to the longitudinal, expert, all-data (LEAD) standard [27], [28]. Table 1 shows the prevalence of the different PD diagnoses in the sample. Additional details regarding sociodemographic and diagnostic characteristics are reported by Pedersen and Karterud [29]. All participants (n = 546) filled out the ANPS. Some missing items occurred within the total sample, but due to the low frequency and lack of systematic pattern, they were regarded as random and of no threat to the validity of the inferences from the study.

**Table 1:** Prevalence of PDs in the patient sample (n = 546).

	Total	Males	Females
	n (%)	n (%)	n (%)
Schizoid	1 (0.2)	1 (0.8)	0 (0.0)
Schizotypal	5 (0.9)	2 (1.6)	3 (0.7)
Paranoid	45 (8.3)	15 (12.1)	30 (7.1)
Borderline	211 (38.8)	35 (28.2)	176 (41.9)
Antisocial	3 (0.6)	1 (0.8)	2 (0.5)
Narcissistic	18 (3.3)	10 (8.1)	8 (1.9)
Histrionic	2 (0.4)	1 (0.8)	1 (0.2)
Avoidant	149 (27.4)	38 (30.6)	111 (26.4)
Dependent	25 (4.6)	5 (4.0)	20 (4.8)
Obsessive-Compulsive	38 (7.0)	14 (11.3)	24 (5.7)
PD NOS	104 (19.1)	27 (21.8)	77 (18.3)
No PD	88 (16.2)	16 (12.9)	72 (17.1)

*Note*. PD = Personality disorder; NOS = Not otherwise specified.

### Ethics

All data from the different hospitals were collected in a central, anonymous database, administrated by the Department for Personality Psychiatry, Oslo University Hospital. All patients provided written consent to participate in the research. All participants in the study were tested for personality functioning, and none was found to have a comprised capacity for consent. The State Data Inspectorate and the Regional Committee approved the procedures for Medical Research and Ethics.

### Assessment

The Norwegian translation of the ANPS was created in accordance with the guidelines of Hambleton [30] by a group of eight clinicians and researchers. The final translation was retranslated back to English by a bilingual translator, and then compared with the original questionnaire.

The 112 items of the ANPS [15] are formed as statements, such as: “When I am frustrated, I usually get angry” and “I often feel sad”. The statements are answered on a 4-point Likert scale [31]: ‘Strongly disagree’, ‘Disagree’, ‘Agree’, and ‘Strongly agree’. As noted above, the ANPS has been developed to reflect individual variation in six basic emotional tendencies. All scales (See File S1 for definitions) are composed by 14 items (See File S2). The scoring procedure for ANPS is to arrange the item scores from 0–3 [14]. Then, adding the item scored gives a range of scale scores from 0–42.

All items for the short form ANPS-S [2] were derived from ANPS. Since all scales comprised of six items, the possible range of the scales are 0–18, and the sum scores are thus not directly comparable to the ANPS scale scores comprising 14 items each (See File S3).

The brief form BANPS [3] kept 28 items from the original ANPS and added five new items (See File S4). Two of these were indicators of SADNESS, two were indicators of SEEK, and one was indicator of CARE. In their two first sample studies Barrett and colleagues [3] coded the responses on a scale ranging from 1–4. Therefore, before the computations of BANPS mean scale scores in this study, the respective items were re-coded from a 0–3 format into a 1–4 format. This will only increase the mean scale scores by 1.0, making them comparable with the findings from the two first sample studies of Barrett and colleagues [3]. In this study we have computed the BANPS scores by the 28 items retrieved from the original ANPS. Thus, in the BANPS part of this study, PLAY consisted of six items, SEEK consisted of four items (out of six), CARE consisted of three items (out of four), FEAR consisted of five items, ANGER consisted of six items, and SADNESS consisted of four items (out of six). According to classical test theory, the primary emotions are regarded as latent variables, all items are assumed as equal indicators of their respective constructs, and the variance of these latent variables are assumed to cause variance of their indicators. By this, a computation of mean scores from the items/indicators of SEEK, CARE and SADNESS should, theoretically, give the same average scale score and variation, even with a lack of two, one, and two items respectively. However, this assumption rests on its empirical support. Therefore, the analysis of the BANPS in this study cannot be regarded as a fully adequate replication of Barrett and colleagues study [3].

### Statistics

By use of IBM SPSS Statistics for Windows, Version 19.0 [32], internal consistency was estimated by Cronbach’s alpha [23], group differences were analyzed by Independent Samples *t*-test (two-sided) and linear relationships between variables were estimated by Pearsons Product Moment Correlations. Exploratory factor analysis was conducted by Principal Axis Factoring (PAF) with Promax rotation. Effect sizes of gender differences were estimated by Hedge’s g [33]. Confirmatory factor analysis (CFA) was conducted with Mplus 7.11 [34]. The items of ANPS was scored on a four-point Likert scale, and therefore regarded as ordered categorical. Therefore, estimations were based on the Mean- and Variance-adjusted Weighted Least Squares (WLSMV) function [35].

To evaluate the CFA models, goodness of fit were estimated by Root Mean Square Error of Approximation (RMSEA) [36], the Non-Normed Fit Index (NFI) [37] also called the Tucker Lewis index (TLI) [38], the Comparative Fit Index (CFI) [39], and the Weighted Root Mean Square Residual (WRMR) [40].

When large samples are used, the Chi-Square statistic nearly always rejects the model [41]. When small samples are used, power decreases and the Chi-Square statistic may fail to discriminate between well and poorly fitted models [42]. Browne and Cudeck [43] proposed a number of measures accounting for the error of approximation and for the precision of the measure itself. One of these population discrepancy functions is the RMSEA [36], which measures the discrepancy per degree of freedom. A RMSEA of 0.05 or below indicates good model fit, values between 0.05 and 0.08 indicate a reasonable fit, between 0.08 and 0.10 a mediocre fit, and above 0.10 a non-acceptable fit [44]. However, a cut-off value close to 0.06 [40] or a stringent upper limit of 0.07 [45] seems to be the general consensus. The TLI and the CFI both measure model fit as compared to the independence model. Both are derived from the chi square statistic, and are supposed to lie between 0 and 1. Values greater than 0.90 for these measures are normally required for good fit of a model, although Hu and Bentler [40] have suggested TLI≥0.95 as the threshold. According to Yu [40], a value < = 1.0 for WRMR is indicative of good model fit.

A visual evaluation of the unidimensionality of the scales was given by Cronbach-Mesbah curves [46] generated by the R Package CMC [47] of the open-source R programming environment [48]. This is also known as the Backward Cronbach Alpha Curve [49].

From the Spearman-Brown formula Cronbach’s alpha [23] is an increasing function of the number of variables. Then, a step-by-step curve of alpha can be built to assess the one-dimensionality of a set of variables. The first step uses all variables to compute the total alpha of the scale. Then, at every successive step, one variable is removed. The removed variable is the one that leaves the scale with its maximum alpha value, and the procedure is repeated until only two variables remain. Then, the alpha value after each removed item is plotted on a curve in a reversed order of removement. If the increased number of variables increases the reliability of the total score, then unidimensionality is supported. A decrease of the curve after adding a variable would indicate that the added variable did not constitute a unidimeninsional set with the other variables.

## Results

### Scale Scores and Internal Consistency of ANPS

The observed mean scores on the original ANPS scales ranged from about 21 to 30 (Table 2). Gender differences were observed on the CARE, FEAR, and SADNESS subscales, in which men scored significantly lower than women. Based on the effect sizes (Hedge’s g) differences were small to moderate [50].

**Table 2:** Mean levels, internal consistency, and gender differences in the ANPS scales.

	Total sample		Cronbach’s alpha	Men		Women		Gender	Hedge’s
	(n = 546)		(95% CI; MIIC)	(n = 124)		(n = 422)		diff.*	g
	Mean	SD		Mean	SD	Mean	SD		
PLAY	22.43	6.73	.84 (.82–.86; .28)	21.74	7.28	22.63	6.55	ns	0.15
SEEK	21.48	6.49	.82 (.79–.84; .25)	21.64	5.75	21.43	6.70	ns	0.04
CARE	27.89	5.86	.74 (.70–.77; .17)	24.56	5.77	28.86	5.52	p<.001	0.79
FEAR	29.16	6.57	.84 (.82–.86; .28)	27.56	6.34	29.63	6.58	p<.01	0.32
ANGER	23.05	7.80	.87 (.85–.88; .31)	22.21	7.56	23.29	7.86	ns	0.13
SADNESS	29.30	5.68	.75 (.71–.78; .18)	26.89	5.63	30.01	5.50	p<.001	0.56

Note. MIIC = Mean Inter-Item Correlation within scale. *) Independent Samples t-test (two-sided).

Cronbach’s alpha values ranged from .75 (SADNESS) to .87 (ANGER). According to the corrected item-total correlations, some items appeared less suitable as indicators of their respective construct. This means that their correlation with the scale, without the item itself, is in the low range of 0.0–0.3, indicating that the question is not discriminating well. This was true for item 9 from the SEEK scale, items 11, 19 and 43 from the CARE scale, item 58 from the FEAR scale, item 12 from the ANGER scale, and items 14, 46, and 110 from the SADNESS scale. In addition, several scales contained items asking the same question, only with reversed wording. These were item 25 and 105 of SEEK, item 19 and 43, and 35 and 59 from CARE, item 66 and 90 of FEAR, item 4 and 44 of ANGER, and item 22 and 78 of SADNESS (see Appendix B). The mean inter-correlations of items within each scale ranged from 0.17 (CARE) to 0.31 (ANGER).

**Figure 1:**
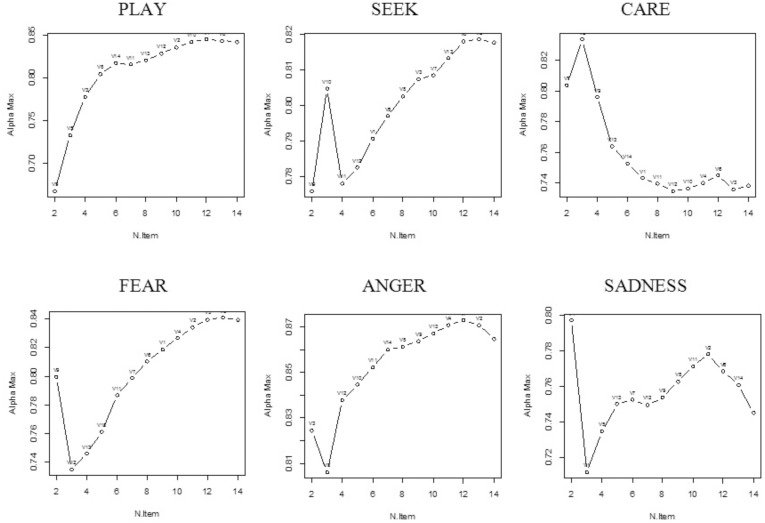
Backward Cronbach Alpha Curves of ANPS scales.

The Backward Cronbach Alpha (BCA) Curves visualize some weakness of all ANPS scales (Figure 1). PLAY, SEEK, FEAR and ANGER indicates a potential for improvement, whereas the plot of alphas for CARE and SADNESS clearly indicate a serious lack of unidimensionality.

Correlations between the six ANPS scale scores ranged from −0.28 (SEEK and FEAR) to 0.59 (FEAR and SADNESS), indicating low to moderate linear relationships (Table 3).

**Table 3:** Correlations between ANPS scales.

	PLAY	SEEK	CARE	FEAR	ANGER
SEEK	.50***				
CARE	.39***	.24***			
FEAR	–.22***	–.28***	.16***		
ANGER	.01	.14**	–.03	.22***	
SADNESS	–.06	–.07	.36***	.59***	.25***

Note: Significance level: ***) p<.001, **) p<.01, *) p<.05 (2-tailed), Pearson product-moment correlation.

### Scale Scores and Internal Consistency for BANPS

The observed mean scores of BANPS ranged from 2.44 (ANGER) to 3.24 (FEAR). Gender differences were observed on PLAY, CARE, FEAR, and SADNESS, in which men scored lower than women. As with the gender differences observed on the scales on ANPS, these were also small to moderate (Table 4).

**Table 4:** Mean levels, internal consistency, and gender differences of BANPS^1^.

	Total sample		Cronbach’s alpha	Men		Women		Gender	Hedge’s
	(n = 546)		(95% CI; MIIC)	(n = 124)		(n = 422)		diff.*	g
	Mean	SD		Mean	SD	Mean	SD		
PLAY	2.81	0.59	.80 (.77–.83; .40)	2.67	0.65	2.84	0.56	p<.01	0.29
SEEK	2.69	0.63	.73 (.69–.77; .41)	2.69	0.58	2.69	0.65	ns	0.00
CARE	2.81	0.66	.61 (.54–.66; .34)	2.66	0.66	2.85	0.65	p<.01	0.29
FEAR	3.24	0.55	.75 (.71–.78; .38)	3.07	0.57	3.30	0.53	p<.001	0.43
ANGER	2.44	0.66	.80 (.77–.82; .40)	2.39	0.61	2.46	0.68	ns	0.11
SADNESS	3.23	0.56	.70 (.66–.74; .38)	3.06	0.55	3.28	0.55	p<.001	0.40

*Note*. 1) Mean scores ranges from 1–4. MIIC = Mean Inter-Item Correlation within scale. *) Independent Samples *t*-test (two-sided).

Cronbach’s alpha values ranged from .61 (CARE) to .80 (PLAY/ANGER). According to the corrected item-total correlations, all items appeared as equally representative indicators of their respective construct. The mean inter-correlations of items within each scale ranged from 0.34 (CARE) to 0.40 (PLAY/ANGER).

**Figure 2:**
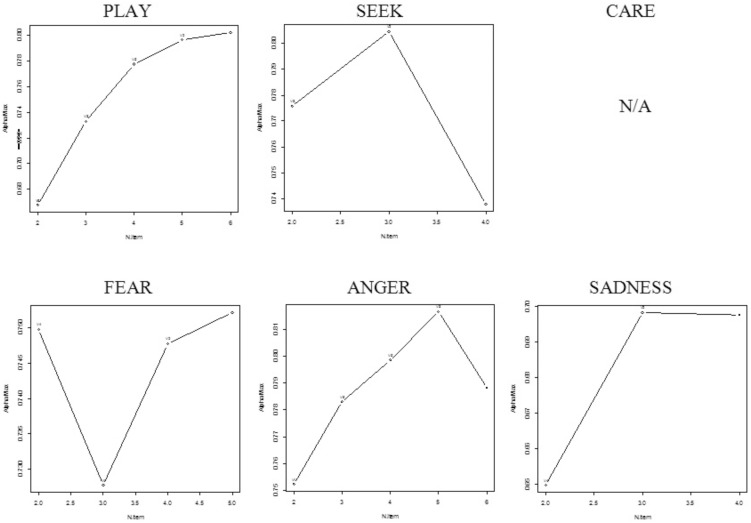
Backward Cronbach Alpha Curves of BANPS scales.

The BCA Curves of BANPS (Figure 2) also indicates some challenge with respect to unidimensionality. However, in this study CARE comprised only three items, which is too few for a BCA Curve. SEEK and SADNESS, with four items each, could technically be plotted although interpretations must be done with care. PLAY revealed a clear unidimensional pattern, whereas FEAR and ANGER seems to have a potensial for improvement.

Correlations between the BANPS scale scores ranged from −0.10 (PLAY/FEAR) to 0.49 (FEAR and SADNESS), indicating low to moderate linear relationships.

### Scale Scores and Internal Consistency for ANPS-S

Observed ANPS-S scale scores ranged from about 9 (ANGER) to 13 (SADNESS). Gender differences were observed on CARE, FEAR, ANGER, and SADNESS, in which men scored lower than women. Again, differences were small to moderate (Table 5).

**Table 5:** Mean levels, internal consistency, and gender differences in the ANPS-S.

	Total sample		Cronbach’s alpha	Men		Women		Gender	Hedge’s
	(n = 546)		(95% CI; MIIC)	(n = 124)		(n = 422)		diff.*	g
	Mean	SD		Mean	SD	Mean	SD		
PLAY	10.02	3.26	.72 (.69–.76; .31)	9.77	3.29	10.09	3.25	ns	0.10
SEEK	9.99	3.45	.78 (.75–.81; .37)	10.07	3.02	9.97	3.56	ns	0.03
CARE	11.75	3.10	.65 (.60–.70; .23)	10.71	2.75	12.05	3.14	p<.001	0.44
FEAR	11.12	3.58	.76 (.73–.80; .36)	10.23	3.16	11.38	3.66	p<.001	0.32
ANGER	9.27	4.16	.84 (.82–.86; .46)	8.48	3.88	9.51	4.22	p<.01	0.25
SADNESS	13.03	3.00	.69 (.64–.73; .28)	12.01	3.00	13.33	2.94	p<.001	0.45

*Note*. MIIC = Mean Inter-Item Correlation within scale. *) Independent Samples *t*-test (two-sided).

Cronbach’s alpha values ranged from .65 (CARE) to .84 (ANGER). According to the corrected item-total correlations, no items deviated substantially from their scales, and the mean inter-correlations of items within each scale ranged from 0.23 (CARE) to 0.46 (ANGER).

**Figure 3:**
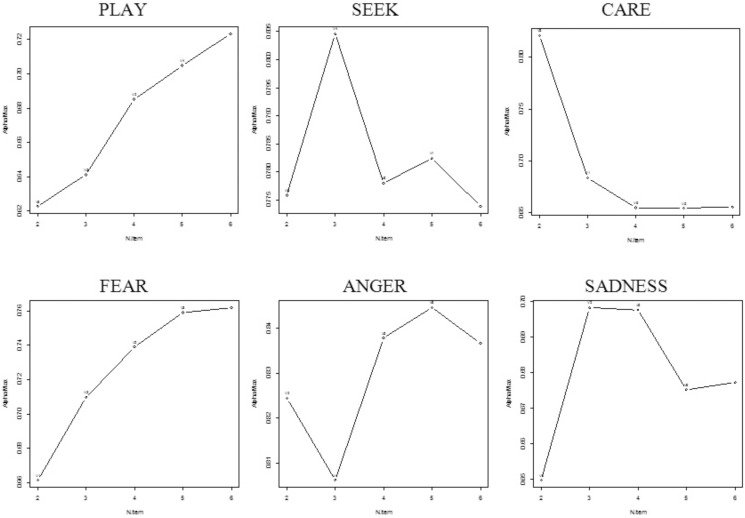
Backward Cronbach Alpha Curves of ANPS-S scales.

BCA Curves of the ANPS-S scales (Figure 3) indicates that only PLAY and FEAR represents a unidimensional set of indicators, whereas the rest of the scales indicates serious challenges, especially SEEK, CARE and SADNESS.

Correlations between the ANPS-S scale scores ranged from −0.19 (PLAY and FEAR) to 0.43 (PLAY and SEEK), also indicating low to moderate linear relationships (Table 6).

**Table 6:** Correlations between BANPS−/ANPS-S scales.

	PLAY	SEEK	CARE	FEAR	ANGER	SADNESS
PLAY	-	.34***	.34***	–.10*	.02	–.11*
SEEK	.43***	-	.24***	–.06	.21***	–.04
CARE	.26***	.19***	-	.09*	.00	.03
FEAR	–.19***	–.20***	.09*	-	.16***	.49***
ANGER	.02	.14***	–.09*	.09	-	.21***
SADNESS	–.08	–.02	.17***	.41***	.21***	-

*Note:* BANPS scales above diagonal, ANPS-S scales below diagonal. Significance level: ***) p<.001, **) p<.01, *) p<.05 (2-tailed), Pearson product-moment correlation.

Not surprisingly, the six primary emotions as operationalized by ANPS, BANPS and ANPS-S were highly correlated (Table 7). However, there were some minor exceptions due to slightly different focus on the different aspects of the emotions, as measured by ANPS. This is most explicit for the operationalization of CARE.

**Table 7:** Correlations between ANPS, BANPS, and ANPS-S.

	PLAY	SEEK	CARE	FEAR	ANGER	SADNESS
ANPS with:						
BANPS	.89	.80	.62	.88	.93	.72
ANPS-S	.93	.90	.76	.85	.91	.86
BANPS with:						
ANPS-S	86	.83	.44	.72	.94	.89

*Note:* All correlations significant at the .001 level (2-tailed), Pearson product-moment correlation.

### Confirmatory factor analysis of ANPS, BANPS and ANPS-S

According to conventional interpretations of Goodness of Fit statistics, neither ANPS nor BANPS or ANPS-S revealed good fit to a six-factor model of the primary scales. However, based on the fit indices, BANPS and ANPS-S revealed better fit to a six-factor model than ANPS, with BANPS revealing the best fit (Table 8). According to their levels of TLI [37] and CFI [39], both of these short-versions can certainly be improved.

**Table 8:** Goodness of Fit Statistics from CFA’s.

	Chi-Square	df	RMSEA (90% CI)	TLI	CFI	WRMR
Six-Factor Models:						
ANPS	7828.18	3387	0.049 (0.048–0.050)	0.752	0.759	2.112
BANPS	852.09	335	0.053 (0.049–0.058)	0.924	0.933	1.364
ANPS-S	1844.39	579	0.063 (0.060–0.067)	0.851	0.863	1.798

*Note:* Chi-Square statistics: *p* values<0.0001.

### Exploratory Factor Analysis of ANPS

Based on the 84 items from the six primary scales of the ANPS, the Kaiser-Meyer-Olkin Measure of Sampling Adequacy was .80, indicating that the correlation matrix was adequate for factor analysis [51], [52]. A principal axis factoring (PAF) with Promax rotation converged with 23 factors accounting for 66% of the variance. By ignoring items with factor loadings <0.3, the first four factors were defined by items from ANGER, PLAY, FEAR and SEEK, respectively. Factor five was defined by items from SEEK and PLAY, factor six by items from CARE, and factor seven by items from SADNESS and CARE (Table 9). The remaining 16 extracted factors were all defined by less than three items each when the limits for interpretation of factor loadings were set to 0.3. The first seven factors accounted for 40% of the variance.

**Table 9:** PAF of the ANPS primary scale items.

First seven extracted factors							
(Explained variance) [Eigenvalues]							
	1	2	3	4	5	6	7
	(11.4%)	(9.9%)	(6.4%)	(3.6%)	(3.2%)	(2.8%)	(2.7%)
	[9.54]	[8.33]	[5.40]	[3.01]	[2.69]	[2.34]	[2.24]
Item:	4 (ANGER)	5 (PLAY)	2 (FEAR)	1* (SEEK)	9 (SEEK)	11 (CARE)	54 (SADN)
	20 (ANGER)	13 (PLAY)	10 (FEAR)	25 (SEEK)	17 (SEEK)	35 (CARE)	70* (SADN)
	28 (ANGER)	21 (PLAY)	42 (FEAR)	33* (SEEK)	41 (SEEK)	51 (CARE)	94 (SADN)
	36 (ANGER)	37 (PLAY)	58 (FEAR)	65 (SEEK)	49* (SEEK)	59 (CARE)	99* (CARE)
	44 (ANGER)	53 (PLAY)	74 (FEAR)	73 (SEEK)	93* (PLAY)		102 (SADN)
	60 (ANGER)	61 (PLAY)	98 (FEAR)	81 (SEEK)	101* (PLAY)		
	68* (ANGER)	69 (PLAY)		89 (SEEK)			
	76 (ANGER)	77 (PLAY)		105 (SEEK)			
	84 (ANGER)						
	92 (ANGER)						
	100* (ANGER)						
	108* (ANGER)						

*Note:* Chi-Square statistics: *p* values<0.0001.

## Discussion

This is the first study to investigate the psychometric properties of the ANPS in a clinical sample of individuals with PDs. In addition we have compared the ANPS with two different short versions. For the full ANPS, the main findings were as follows: 1) Internal consistencies of the ANPS subscales were acceptable in general, as indicated by Cronbach’s alpha. 2) Several items revealed questionable face validity and poor psychometric properties based upon item-total correlations. In particular this concerned some items from SADNESS and CARE. 3) Correlations between the subscales SADNESS and FEAR as well as PLAY and SEEK were (too) high (.50 and .59). 4) Confirmatory factor analyses at item level revealed poor fit to a six-factor solution. 5) Exploratory factor analyses of ANPS resulted in seven interpretable factors, five of which were analogue to the purposed primary emotions (ANGER, PLAY, FEAR, SEEK, CARE), while two were defined by combinations of items from SEEK/PLAY and SADNESS/CARE, respectively. 6) An explorative factor analysis (PAF) revealed well defined factors of ANGER, PLAY, FEAR and SEEK, while the factors containing CARE and SADNESS items consisted of few items and explained little of the variance.

For the short versions, the main findings were as follows: 1) The internal consistencies of both short versions were acceptable, although bordering on a low level for CARE (.61 for BANS and .65 for ANPS-S). 2) The intercorrelations of PLAY and SEEK, on the one hand, and SADNESS and FEAR were lower for both short versions (.36 and .49 versus .43 and .41). 3) The subscale intercorrelations between all versions of ANPS should optimally be high. The CARE subscale revealed consistently the lowest intercorrelation. 4) A confirmatory factor analysis of the short versions displayed better fit to a six factor model than the full ANPS on a range of indices. Best results were obtained by the BANPS.

In the following we will discuss these findings, concentrating on the major shortcoming of the different versions of the ANPS, which seems to reside in the operationalization of the CARE and SADNESS subscales.

### Operationalization and internal consistency

The subscales of the full ANPS consist of 14 items each. Davis and colleagues [1] stated that their intention was eventually to reduce the number of items to ten per scale. As many as 14 items will usually generate acceptable alpha values, despite several near zero correlations among the items. An interpretation of such scale scores on individual levels are certainly troublesome, e.g. scores on CARE can be low if the respondent does not care for children or pets, even if there is a great need for closeness to others. The short forms have demonstrated that acceptable, or even better, psychometric properties can be obtained by fewer items, e.g. around six. However, by fewer items it is all the more important that the items are close to the essence of the constructs and that they cover different aspects of the constructs, not merely being opposites (like for the SADNESS scale: “I rarely become sad” and “I often feel sad”).

When the operationalizations of the constructs become narrower, there is a risk that different short versions cover different aspects of the constructs. Most different in this study are the CARE scales of the two short forms, with a correlation of just .46. The most apparent reason for this is that BANPS only address needs for closeness, whereas ANPS-S also includes items addressing care for children. Another difference between the short versions is on FEAR, wherein ANPS-S includes items addressing general concerns, whereas BANPS assess worries. Contrary to ANPS-S, the SADNESS scale of BANPS does not address concerns about separation from home and friends, which also reduces its linear relationship with the original ANPS.

Face validity of the different items depends on the closeness to the essence of the constructs. For the subscale of SADNESS we note a deviance from the original primary emotion already by the label. The primary emotion underlying sadness is not sadness per se, but SEPARATION DISTRESS/PANIC [53]. When we inspect the content of the 6 SADNESS items of the BANPS, we find that they cover only two aspects, namely “I often/seldom feel sad” and “I often/seldom feel lonely”. For these construct validity reasons alone, the BANPS SADNESS subscale should be revised by deleting redundant items and replacing them with some more appropriate items addressing the biological (and evolutionary) underpinning of the construct (i.e. separation distress).

Concerning the subscale CARE, the full ANPS contain as much as four items that concern affectionate feelings towards pets, in particular young dogs and cats. These four items are the same as those found in factor 6 of the exploratory factor analysis (Table 9). These items obviously correlate highly among themselves, but low with the other items of CARE. The BANPS has “solved” this problem by deleting all pet items. The problem with the CARE subscale of the BANPS is that it is reduced to four items, and that these items cover only two aspects, namely 1) being or not being affectionate, and 2) wanting or not wanting closeness to others.

This brings us back to the original primary emotion construct of CARE [53]. It is conceived, as the other primary emotions, in evolutionary terms, being valid for all mammals, to cover primarily maternal care and affection for the offspring, linked to attachment and attachment behavior, e.g. responding to separation/fear distress calls. Among higher primates it is linked to empathy and among Homo sapiens also to (non-sexual) love and affection extending beyond own children. The two aspects covered by BANPS should be supplemented with four items covering the themes mentioned above.

### Gender Differences

Gender differences were as expected, both from an evolutionary perspective and in accordance with previous studies. Women rated higher on CARE, FEAR, and SADNESS on all versions. However, by BANPS females also scored higher than men on PLAY, and by ANPS-S they scored higher than men on ANGER. One could consider the last finding to be a peculiarity of this sample, consisting of a high proportion of (angry) borderline women. However, in the Canadian sample of Pingault and colleagues [2], females (with comparable age) also scored higher than males on ANGER, as well as on CARE, FEAR and SADNESS.

### Intercorrelations between scales

We have found, consistent with previous research, that SEEK and PLAY have a substantial intercorrelation (.50 for ANPS) as well as SADNESS and FEAR (.59 for ANPS). Do these intercorrelations reflect a true state of human nature or are they artefacts due to poor item validity? The fact that the intercorrelations are weaker for ANPS-S and BANPS, indicates that at least a portion of the confluence is due to measurement error because of poor item validity. In theory, the different primary emotions are believed to have evolved as distinct (modular) responses to crucial environmental challenges. Positive correlations among some of these subscales imply that if one has an inclination/low threshold for e.g. SEEK, one also has an inclination for PLAY. An indication for that in fact being true, could be the fact that the extroversion factor in the Five Factor Model is largely composed of the primary emotions SEEK and PLAY [19]. It is reasonable to suppose that in order to play, one has to engage the SEEK system. This is not the case with e.g. the FEAR system. FEAR is known to shut down the systems of SEEK, PLAY, CARE and LUST, and even ANGER if the danger is perceived as overwhelming. However, FEAR is also known to trigger SEPARATION anxiety and vice versa. Accordingly there are good arguments for these positive correlations being a reflection of nature. On the other hand, such a natural connection may be exaggerated by conceptual ambiguities. The kind of fear being activated by SEPARATION DISTRESS, may be difficult to disentangle from fear of dangerous situations and objects. It is a target for future research to clarify these issues. In any case we should be careful to ensure that the items chosen for each subscale are closely related to the essence of the particular primary emotion.

### Factor Analyses

The confirmatory factor analyses revealed that the BANPS almost possessed a good fit to a six-factor model, that the ANPS-S did poorer than BANPS, and that the full ANPS fell short of a six-factor model. As noted, several of the ANPS scales had items with rather low levels of mean inter-item correlations, and this will affect the incremental fit indexes such as TLI and CFI. With respect to BANPS it is important to keep in mind that the analysis is conducted on a truncated version.

With reference to their psychometric properties both BANPS and ANPS-S passed as more coherent instruments than ANPS, although BANPS seems to be the best based on data from the current patient sample. However, the current short versions should be regarded as way stations to even more effective and valid assessment of the six primary emotions. The factor analysis of ANPS in the current sample supports only partly the operationalizations of the short versions. That is, items comprised by ANGER, PLAY, and SEEK are much the same between the three solutions. However, none of them is measuring CARE by the same items, and BANPS measures FEAR somewhat differently than ANPS-S and the items in the factor solution of ANPS on the current sample. Furthermore, both short-versions measure SADNESS by mostly the same items, but in the current sample these items did not appear as indicators of a common factor.

The main purpose of the EFA was to explore the pattern of factor loadings. As it turned out, the content validity of the last three factors was quite questionable. Since this study was not meant to purpose any new short form or specific suggestions to item revisions, correlated residuals for items with very similar content are not discussed. The result of the EFA is only meant to illustrate the most challenging part of ANPS.

### Limitations

Most psychometric studies have limitations due to sample peculiarities. The size of the sample in this study was appropriate for the statistical methods employed, i.e. the factor analyses. Earlier studies have been performed on normal populations, in particular young college students. This sample consisted of personality disordered patients, which implies that the range of emotional experiences among the respondents was larger than in previous samples for emotions like FEAR, ANGER, CARE and SADNESS. Also within such a sample we found results that were more or less similar to previous studies.

The main limitation of this study is that psychometric analyses of BANPS were performed on 28 items, and not the full 33 item version, since five items in BANPS were created anew and was not available to us when we conducted the study. Three of these items concerned SADNESS and CARE. The two SADNESS items were already represented by a reverse or similar item, while the CARE item added a new aspect (“I often feel the urge to nurture those closest to me”). We cannot rule out the possibility that these five items would have enhanced the psychometric properties to a level that would have satisfied the requirements of a six factor model. Nor can we rule out the possibility that they would have worsened the instrument. Thus, interpretations of the findings concerning BANPS must be considered with caution, especially with respect to the CFA and the SEEK, CARE, and SADNESS subscales.

A second limitation concerns the extraction of short-forms from original forms. In the current study all participants answered the original 112-item ANPS, and ANPS-S and a truncated version of BANPS were computed from this. We cannot rule out the possibility that the short-forms, administered as separate questionnaires, would have given somewhat different results [54].

### Considerations for further research

It is important to acknowledge that the ANPS is a suggestion to the assessment of the primary emotions as defined by neuroaffective research and theory [12]. When such instruments fails according to conventional psychometric standards it can most often be explained by a too strict model, by latent constructs that are too broadly defined, by weakness in the operationalization of the latent constructs, or by different variance and covariance of indicators across different samples of subjects. The scales of ANPS are broadly defined by several indicators addressing somewhat different aspects of each construct. To resolve problems caused by this diversity by creating short-forms, may enhance the psychometric properties, but at the same time delimit what is being measured. As we have seen, different suggestions to such short-forms might not measure the same latent constructs in the same way. We would therefore suggest that further development of instruments for the assessment of primary emotions should look carefully into the conceptualization and definition of the six primary emotions, and also include the (omitted) emotion of LUST. Special considerations should be given to the operationalizations of CARE and SADNESS, and particularly CARE. At current, these two constructs are poorly assessed by the ANPS and the two short forms.

## Conclusion

Primary emotions are crucial phenomena for a proper understanding of personality and personality disorders. The ANPS was designed as a means to measure primary emotions by a self-report questionnaire. This study on a clinical sample of PD individuals has confirmed previous research findings of psychometric shortcomings, e.g. that the items of ANPS do not fit a six-factor model. Two short forms, ANPS-S and a truncated BANPS, proved to have better psychometric properties. However, there are still potentials for improvement, in particular for the items representing CARE and SADNESS. The ANPS, long and short versions, represent valuable tools to assess primary emotions. However, further work is needed. In further efforts to improve the ANPS, careful theoretical considerations of scale operationalizations should be done. When new suggestions are to be analyzed, this should include samples from both clinical and non-clinical samples.

## Supporting Information

File S1 **Definitions of the ANPS scales.**
(DOC)

File S2 **ANPS Scale Operationalizations.**
(DOC)

File S3 **ANPS_S Scale Operationalizations.**
(DOC)

File S4 **BANPS Scale Operationalizations.**
(DOC)

## References

[1] Davis KL, Panksepp J, Normansell L (2003) The Affective Neuroscience Personality Scales: Normative Data and Implications. Neuropsychoanalysis, 5, 57–69.

[2] Pingault J-B, Falissard B, Côté S, Berthoz S (2012) A New Approach of Personality and Psychiatric Disorders: A Short Version of the Affective Neuroscience Personality Scales. PLoS ONE 7(7), e41489. doi:10.1371/journal.pone.0041489. 10.1371/journal.pone.0041489 PMC3406066 22848510

[3] Barrett FS, Robins RW, Janata P (2013) A Brief Form of the Affective Neuroscience Personality Scales. Psychological Assessment, 25, 826–843. 10.1037/a0032576 23647046

[4] Jang KL, McCrae RR, Angleitner A, Riemann R, Livesley WJ (1998) Heritability of facet level traits in a cross-cultural twin sample: support for a hierachical model of personality. Journal of Personality and Social Psychology, 74, 1556–1565. 10.1037//0022-3514.74.6.1556 9654759

[5] Reichborn-Kjennerud T (2008) Genetics of personality disorders. Psychiatric clinics of North America, 31, 421–440. 10.1016/j.psc.2008.03.012 18638644

[6] Rothbart MK, Ahadi SA, Evans DE (2000) Temperament and personality: origins and outcomes. Journal of Personality and Social Psychology, 78, 122–135. 10.1037//0022-3514.78.1.122 10653510

[7] Darwin C (1872/1998) The expression of emotions in man and animals (3rd edn). New York: Oxford University Press.

[8] Panksepp P (2012) What is an emotional feeling? Lessons about affective origins from cross-species neuroscience. Motivation and Emotion, 36, 4–15.

[9] Tomkins SS (1962) Affect imagery consciousness: Vol. I. The positive affects. New York: Springer.

[10] Tomkins SS (1963) Affect imagery consciousness: Vol. II. The negative affects. New York: Springer.

[11] Damasio AR (1999) The feeling of what happens: Body and emotion in the making of consciousness. New York: Harcourt.

[12] Panksepp J (1998) Affective Neuroscience: The Foundations of Human and Animal Emotions. New York: Oxford University Press.

[13] Panksepp J (2007) Criteria for basic emotions: Is DISGUST a primary “emotion”? Cognition and Emotion, 21, 1819–1828.

[14] Spielberger C D (1975) The measurement of state and trait anxiety: Conceptual and methodological issues. In: Emotions: Their Parameters and Measurement, ed. L Levi. New York: Raven Press.

[15] Davis KL, Panksepp J (2011) The brain’s emotional foundations of human personality and the Affective Neuroscience Personality Scales. Neuroscience & Biobehavioral Reviews, 35, 1946–1958. 10.1016/j.neubiorev.2011.04.004 21527289

[16] Kendler KS, Liu X-Qi, Gardner CO, McCullough ME, Larson D, Prescott CA (2003) Dimensions of religiosity and their relationship to lifetime psychiatric and substance use disorders. American Journal of Psychiatry, 160, 496–503. 10.1176/appi.ajp.160.3.496 12611831

[17] Miller WR, Thoresen CE (2003) Spirituality, religion, and health: An emerging research field. American Psychologist, 58, 24–35. 10.1037/0003-066x.58.1.24 12674816

[18] Panksepp J, Nocjar C, Burgdorf J, Panksepp JB, Huber R (2004) The role of emotional systems in addiction: a neuroethological perspective. In: Bevins, R.A., Bardo, M.T. (Eds.), 50th Nebraska Symposium on Motivation: Motivational Factors in the Etiology of Drug Abuse. Nebraska, Lincoln. 85–126. 15160639

[19] Costa PT, McCrae RR (1992) Revised NEO Personality Inventory (NEO-PI-R) and NEO Five factor Inventory (NEO-FFI), Professional Manual. Odessa (FL): Psychological Assessment Resources, Inc.

[20] Pahlavan F, Mouchiroudb C, Zenasnib F, Panksepp J (2008) Validation de l’adaptation française de l’échelle neuro-affective de personnalitéstar. French validation of the Affective Neuroscience Personality Scales (ANPS). Revue Européenne de Psychologie Appliquée/European Review of Applied Psychology, 58, 155–163.

[21] Pingault J-B, Pouga L, Grezes J, Berthoz S (2011) Determination of emotional endophenotypes: A validation of the Affective Neuroscience Personality Scales and further perspectives. Psychological Assessment, 24, 375–385. 10.1037/a0025692 21942230

[22] Abella V, Panksepp J, Manga D, Bárcena C, Iglesias JA (2011) Spanish Validation of the Affective Neuroscience Personality Scales. The Spanish Journal of Psychology, 14, 926–935. 10.5209/rev_sjop.2011.v14.n2.38 22059336

[23] Cronbach LJ (1951) Coefficient alpha and the internal structure of tests. Psycometrika, 16, 297–334.

[24] Karterud S, Pedersen G, Bjordal E, Brabrand J, Friis S, et al.. (2003) Day hospital treatment of patients with personality disorders. Experiences from a Norwegian treatment research network. Journal of Personality Disorders, 17, 173–193. 10.1521/pedi.17.3.243.22151 12839103

[25] American Psychiatric Association (1994) Diagnostic and statistical manual of mental disorders (4th edn). Washington, DC: American Psychiatric Association.

[26] First MB, Gibbon M, Spitzer RL, Williams JBW, Benjamin LS (1997) The Structured Clinical Interview for DSM-IV Axis II Personality Disorders (SCID-II). Washington, DC, American Psychiatric Press.

[27] Pedersen G, Karterud S, Hummelen B, Wilberg T (2013) The impact of extended longitudinal observation on the assessment of personality disorders. Personality and Mental Health, 7, 277–287. 10.1002/pmh.1234 24343977

[28] Spitzer RL (1983) Psychiatric diagnoses: Are clinicians still necessary? Comprehensive Psychiatry, 24, 399–411. 10.1016/0010-440x(83)90032-9 6354575

[29] Pedersen G, Karterud S (2007) Associations between patient characteristics and ratings of treatment milieu. Nordic Journal of Psychiatry, 61, 271–278. 10.1080/08039480701414999 17763120

[30] Hambleton RK (2005) Issues, designs and technical guidelines for adapting test into multiple languages and cultures. In Hambleton RK, Merenda PF, Spielberger SD (Eds.): Adapting educational and psychological test for cross-cultural assessment. Mahwah, NJ: Lawrence Erlbaum Associates. 3–38.

[31] Likert R (1932) A Technique for the Measurement of Attitudes, Archives of Psychology, 140, 1–55.

[32] IBM (2010) IBM SPSS Statistics for Windows, Version 19.0. Armonk, NY: IBM Corp.

[33] Hedges LV (1981) Distribution theory for Glass’s estimator of effect size and related estimators. Journal of Educational Statistics, 6, 107–128.

[34] Muthén LK, Muthén BO (2012) Mplus User’s Guide. Seventh Edition. Los Angeles, CA: Muthén & Muthén.

[35] Flora DB, Curran PJ (2004) An Empirical Evaluation of Alternative Methods of Estimation for Confirmatory Factor Analysis With Ordinal Data. Psychological Methods, 9, 466–491. 10.1037/1082-989X.9.4.466 PMC3153362 15598100

[36] Steiger JH (1990) Structural model evaluation and modification: an interval estimation approach. Multivariate Behavioral Research, 25, 174–180. 10.1207/s15327906mbr2502_4 26794479

[37] Bentler PM, Bonett DG (1980) Significance tests and goodness of fit in the analysis of covariance structures. Psychological Bulletin, 88, 588–606.

[38] Tucker LR, Lewis C (1973) The reliability coefficient for maximum likelihood factor analysis. Psychometrika, 38, 1–10.

[39] Bentler PM (1990) Comparative fit indexes in structural models. Psychological Bulletin, 107, 238–246. 10.1037/0033-2909.107.2.238 2320703

[40] Yu C-Y (2002) Evaluating Cutoff Criteria of Model Fit Indices for Latent Variable Models with Binary and Continuous Outcomes. Doctoral dissertation, University of California, LA.

[41] Jöreskog KG, Sörbom D (2013) LISREL 9.10 for Windows [Computer software]. Skokie, IL: Scientific Software International, Inc.

[42] Kenny DA, McCoach DB (2003) Effect of the Number of Variables on Measures of Fit in Structural Equation Modeling. Structural Equation Modeling, 10, 333–351.

[43] Browne MW, Cudeck R (1993) Alternative ways of assessing model fit. In Bollen KA, Long JS (Eds), Testing Structural Equation Models. Beverly Hills (CA): Sage. 136–162.

[44] MacCallum RC, Browne MW, Sugawara HM (1996) Power Analysis and Determination of Sample Size for Covariance Structure Modeling. Psychological Methods, 1, 130–149.

[45] Steiger JH (2007) Understanding the limitations of global fit assessment in structural equation modeling. Personality and Individual Differences, 42, 893–898.

[46] Mesbah M (2010) Statistical Quality of Life. In Balakrishnan N (Edt.). Method and Applications of Statistics in the Life and Health Sciences, Wiley. 839–864.

[47] Cameletti M, Caviezel V (2013) The R package CMC to calculate the Cronbach-Mesbah curve. Annales de l’I.S.U.P. Institut de statistique de l’Université de Paris, 57, 59–68.

[48] R Development Core Team. R (2010) A Language and Environment for Statistical Computing. R Foundation for Statistical Computing, Vienna, Austria. ISBN 3-900051-07-0, URL http://www.R-project.org.

[49] Curt F, Mesbah M, Lellouch J, Dellatolas D (1997) Handedness Scale: How Many and Which Items? Laterality, 2, 137–154. 10.1080/713754262 15513060

[50] Cohen J (1988) Statistical Power Analysis for the Behavioral Sciences (second ed.). Lawrence Erlbaum Associates.

[51] Dziuban CD, Shirkey EC (1974) When is a correlation matrix appropriate for factor analysis? Psychological Bulletin, 81, 358–361.

[52] Cerny CA, Kaiser HF (1977) A study of a measure of sampling adequacy for factor-analytic correlation matrices. Multivariate Behavioral Research, 12, 43–47. 10.1207/s15327906mbr1201_3 26804143

[53] Panksepp J, Biven L (2012) The archaeology of mind: Neuroevolutionary origins of human emotion. New York: Norton.

[54] Smith GT, McCarthy DM, Anderson KG (2000) On the Sins of Short-Form Development. Psychological Assessment, 12, 102–111. 10.1037//1040-3590.12.1.102 10752369

