# Correction: Testing Different Versions of the Affective Neuroscience Personality Scales in a Clinical Sample

**DOI:** 10.1371/journal.pone.0114449

**Published:** 2014-11-26

**Authors:** The PLOS ONE Staff

The first author's name is displayed incorrectly. The correct name is: Geir Pedersen. The correct citation is: Pedersen G, Selsbakk JM, Theresa W, Sigmund K (2014) Testing Different Versions of the Affective Neuroscience Personality Scales in a Clinical Sample. PLoS ONE 9(10): e109394. doi:10.1371/journal.pone.0109394

## References

[1] Geir P, Selsbakk JM, Theresa W, Sigmund K (2014) Testing Different Versions of the Affective Neuroscience Personality Scales in a Clinical Sample. PLoS ONE 9(10): e109394 doi:10.1371/journal.pone.0109394 10.1371/journal.pone.0109394 PMC4188588 25289939

