# Supplementary material for: Testing Different Versions of the Affective Neuroscience Personality Scales in a Clinical Sample
Source: PLoS One. 2014 Oct 7;9(10):e109394. doi: 10.1371/journal.pone.0109394 (PMC4188588; doi:10.1371/journal.pone.0109394)
Supplement: File S1 — Definitions of the ANPS scales. (DOC) [file pone.0109394.s001.doc]

Supporting Information S1

Definitions of the ANPS scales

PLAY: Having fun versus being serious; playing games with physical contact, humor, and laughter; and being generally happy and joyful.

SEEK: Feeling curious, feeling like exploring, striving for solutions to problems and puzzles, positively anticipating new experiences, and a sense of being able to accomplish almost anything.

CARE: Nurturing, being drawn to young children and pets, feeling softhearted toward animals and people in need, feeling empathy, liking to care for the sick, feeling affection for and liking to care for others, as well as liking to be needed by others.

FEAR: Having feelings of anxiety, feeling tense, worrying, struggling with decisions, ruminating about past decisions and statements, losing sleep, and not typically being courageous.

ANGER: Feeling hotheaded, being easily irritated and frustrated, experiencing anger verbally or physically, and remaining angry for long periods.

SADNESS: Feeling lonely, crying frequently, thinking about loved ones and past relationships, and feeling distress when not with loved ones.
