# Supplementary material for: Testing Different Versions of the Affective Neuroscience Personality Scales in a Clinical Sample
Source: PLoS One. 2014 Oct 7;9(10):e109394. doi: 10.1371/journal.pone.0109394 (PMC4188588; doi:10.1371/journal.pone.0109394)
Supplement: File S2 — ANPS Scale Operationalizations. (DOC) [file pone.0109394.s002.doc]

Supporting Information S2

ANPS Scale Operationalizations

**PLAY:**

5. I am a person who is easily amused and laughs a lot.

13. My friends would probably describe me as being too serious.

21. I am known as one who keeps work fun.

29. I generally do not like vigorous games which require physical contact.

37. I like to joke around with other people.

45. I dislike humour that gets really silly.

53. People who know me would say I am a very fun-loving person.

61. I do not particularly enjoy kidding around and exchanging "wisecracks"

69. I am very playful.

77. I do not tend to see the humour in things many people consider funny.

85. I like all kinds of games including those with physical contact.

93. I do not frequently ask other people to join me for fun activities.

101. I see life as being full of opportunities to have fun.

109. Playing games with other people is not especially enjoyable for me.

**SEEK:**

1. Almost any little problem or puzzle stimulates my interest.

9. I do not get much pleasure out of looking forward to special events.

17. I really enjoy looking forward to new experiences.

25. I am usually not highly curious.

33. Seeking an answer is as enjoyable as finding the solution.

41. I usually feel little eagerness or anticipation.

49. I enjoy anticipating and working towards a goal almost as much as achieving it.

57. I am usually not interested in solving problems and puzzles just for the sake of solving them.

65. My curiosity often drives me to do things.

73. I rarely feel the need just to get out and explore things.

81. Whenever I am in a new place, I like to explore the area and get a better feel for my surroundings.

89. I am not the kind of person that likes probing and investigating problems.

97. I often feel like I could accomplish almost anything.

105. I am not an extremely inquisitive person.

**CARE:**

3. I often feel a strong need to take care of others.

11. I think it is ridiculous the way some people carry on around baby animals.

19. I like taking care of children.

27. Caring for a sick person would be a burden for me.

35. I love being around baby animals.

43. I do not especially like being around children.

51. I feel soft-hearted towards stray animals.

59. I would generally consider pets in my home to be more trouble than they are worth.

67. I feel sorry for the homeless.

75. I do not like to feel "needed" by other people.

83. I am the kind of person that likes to touch and hug people.

91. I do not especially want people to be emotionally close to me.

99. I am a person who strongly feels the pain of other people.

107. I am not particularly affectionate.

**FEAR:**

2. People who know me well would say I am an anxious person.

10. I am not frequently jittery and nervous.

18. I often think of what I should have done after the opportunity has passed.

26. I would not describe myself as a worrier.

34. I often cannot fall right to sleep because something is troubling me.

42. I have very few fears in my life.

50. I sometimes cannot stop worrying about my problems.

58. My friends would say that it takes a lot to frighten me.

66. I often worry about the future.

74. There are very few things that make me anxious.

82. I often worry about whether I am making the correct decision.

90. I rarely worry about my future.

98. I often feel nervous and have difficulty relaxing.

106. I almost never lose sleep worrying about things.

**ANGER:**

4. When I am frustrated, I usually get angry.

12. I never stay irritated at anyone for very long.

20. My friends would probably describe me as hot-headed.

28. I cannot remember a time when I became so angry that I wanted to break something.

36. When I get angry, I often feel like swearing.

44. When I am frustrated, I rarely become angry.

52. When someone makes me angry, I tend to remain fired up for a long time.

60. People who know me well would say I almost never become angry.

68. I tend to get irritated if someone tries to stop me from doing what I want to do.

76. I rarely get angry enough to want to hit someone.

84. When things do not work out the way I want, I sometimes feel like kicking or hitting something.

92. I hardly ever become so angry at someone that I feel like yelling at them.

100. Sometimes little quirky things people do really annoy me.

108. When people irritate me, I rarely feel the urge to say nasty things to them.

**SADNESS:**

6. I often feel sad.

14. I seem to be affected very little by personal rejection.

22. I often have the feeling that I am going to cry.

30. I rarely become sad.

38. I often feel lonely.

46. I never become homesick.

54. I often think about people I have loved who are no longer with me.

62. It does not particularly sadden me when friends or family members are disapproving of me.

70. I tend to think about losing loved ones often.

78. I rarely have the feeling that I am close to tears.

86. I frequently feel downhearted when I cannot be with my friends or loved ones.

94. I rarely think about people or relationships I have lost.

102. I am a person who strongly feels the pain from my personal losses.

110. It would not bother me to spend the holidays away from family and friends.
