# Supplementary material for: Testing Different Versions of the Affective Neuroscience Personality Scales in a Clinical Sample
Source: PLoS One. 2014 Oct 7;9(10):e109394. doi: 10.1371/journal.pone.0109394 (PMC4188588; doi:10.1371/journal.pone.0109394)
Supplement: File S3 — ANPS_S Scale Operationalizations. (DOC) [file pone.0109394.s003.doc]

Supporting Information S3

ANPS-S Scale Operationalizations

**PLAY:**

I am a person who is easily amused and laughs a lot. (5)

I do not particularly enjoy kidding around and exchanging "wisecracks." (61)

I am very playful. (69)

I do not tend to see the humour in things many people consider funny. (77)

I like all kinds of games including those with physical contact. (85)

Playing games with other people is not especially enjoyable for me. (109)

**SEEK:**

I really enjoy looking forward to new experiences. (17)

I am usually not highly curious. (25)

My curiosity often drives me to do things. (65)

I rarely feel the need just to get out and explore things. (73)

Whenever I am in a new place, I like to explore the area and get a better feel for my surroundings. (81)

I am not an extremely inquisitive person. (105)

**CARE:**

I often feel a strong need to take care of others. (3)

I like taking care of children. (19)

Caring for a sick person would be a burden for me. (27)

I do not especially like being around children. (43)

I am a person who strongly feels the pain of other people. (99)

I am not particularly affectionate. (107)

**FEAR:**

People who know me well would say I am an anxious person. (2)

I am not frequently jittery and nervous. (10)

I would not describe myself as a worrier. (26)

I have very few fears in my life. (42)

My friends would say that it takes a lot to frighten me. (58)

There are very few things that make me anxious. (74)

**ANGER:**

When I am frustrated, I usually get angry. (4)

My friends would probably describe me as hot-headed. (20)

When I am frustrated, I rarely become angry. (44)

People who know me well would say I almost never become angry. (60)

I hardly ever become so angry at someone that I feel like yelling at them. (92)

When people irritate me, I rarely feel the urge to say nasty things to them. (108)

**SADNESS:**

I often feel sad. (6)

I often have the feeling that I am going to cry. (22)

I rarely become sad. (30)

I often feel lonely. (38)

I often think about people I have loved who are no longer with me. (54)

I tend to think about losing loved ones often. (70)
