# Supplementary material for: Testing Different Versions of the Affective Neuroscience Personality Scales in a Clinical Sample
Source: PLoS One. 2014 Oct 7;9(10):e109394. doi: 10.1371/journal.pone.0109394 (PMC4188588; doi:10.1371/journal.pone.0109394)
Supplement: File S4 — BANPS Scale Operationalizations. (DOC) [file pone.0109394.s004.doc]

Supporting Information S4

BANPS Scale Operationalizations

**PLAY:**

I am a person who is easily amused and laughs a lot. (5)

My friends would probably describe me as being too serious. (13)

I like to kid around with other people. (37)

People who know me would say I am a very fun-loving person. (53)

I do not particularly enjoy kidding around and exchanging “wisecracks.” (61)

I am very playful. (69)

**SEEK:**

I am usually not highly curious. (25)

I am usually not interested in solving problems and puzzles just for the sake of solving them. (57)

My curiosity drives me to do things. (65)

I am not an extremely inquisitive person. (105)

I like to think outside of the box. (*New seek item*)

I enjoy finding new solutions to problems. (*New seek item*)

**CARE:**

I am the kind of person that likes to touch and hug people. (83)

I do not especially want people to be emotionally close to me. (91)

I am not particularly affectionate. (107)

I often feel the urge to nurture those closest to me. (*New care item*)

**FEAR:**

I have very few fears in my life. (42)

I sometimes cannot stop worrying about my problems. (50)

I often worry about the future. (66)

There are very few things that make me anxious. (74)

I rarely worry about my future. (90)

**ANGER:**

When I am frustrated, I usually get angry. (4)

My friends would probably describe me as hot-headed. (20)

When someone makes me angry, I tend to remain fired up for a long time. (52)

People who know me well would say I almost never become angry. (60)

I rarely get angry enough to want to hit someone. (76)

I hardly ever become so angry at someone that I feel like yelling at them. (92)

**SADNESS:**

I often feel sad. (6)

I often have the feeling that I am going to cry. (22)

I rarely become sad. (30)

I often feel lonely. (38)

I seldom experience sadness or despair. (*New sadness item*)

I do not feel lonely very often. (*New sadness item*)
